# The effects of informed consent format on preoperative anxiety in patients undergoing inferior third molar surgery

**DOI:** 10.4317/medoral.19480

**Published:** 2013-12-07

**Authors:** Daniel Torres-Lagares, Marisa Heras-Meseguer, Francisco Azcárate-Velázquez, Pilar Hita-Iglesias, Gonzalo Ruiz-de-León-Hernández, Esther Hernández-Pacheco, José L. Gutiérrez-Pérez

**Affiliations:** 1 Master in Oral Surgery. University of Seville, Spain; 2 Master in Oral Surgery. University of Michigan, USA

## Abstract

Objectives: To evaluate the effect of informed consent format on preoperative anxiety of patients. Material and Methods: We performed a prospective study (91 patients) undergoing lower third molar extraction. Patients were distributed into three groups. Informed consent for surgery was obtained through a written document, an oral interview or a video recording. Afterwards, patients were asked about their anxiety level and the effect the informed consent had had on it. Results: Whereas the information conveyed both in oral and written formats relieved the patient to some extent (in a scale of -3 to +3) 0.97±1.21 and 0.29±0.97, respectively), the video recording increased patient’s anxiety in a statistically significant way (in a scale of -3 to +3, -0.57±1.43). The difference obtained between the values obtained in oral and written information was not statistically significant. Discussion: The most adequate format, according to our study, would be the oral format.

** Key words:**Anxiety, satisfaction, third molar surgery, Spielberger State-Trait Anxiety Inventory.

## Introduction

Anxiety is an emotional response defined as tension (stress), apprehension, nervousness and concerns caused by an intangible and diffuse advancing threat or approaching danger, accompanied by activation of the autonomous nervous system (1).

Moderate to severe acute postoperative pain occurs frequently after several surgical procedures and affects up to 50% of hospitalized patients and 40% of patients undergoing ambulatory surgery (2).

A point that is far from clear in this respect is the effect the informed consent format has on patient anxiety before surgery.

A good number of authors have studied the effect of anxiety on the experience of pain (measured in both an objective and a subjective way) undergone by patients during and after surgery (2).

Providing patients with relevant information before performing a test or procedure is a fundamental step in the medical practice. The information given to the patient, his family or legal tutor must be conveyed in a clear, precise and understandable language (2).

The main objective of this paper is to evaluate the effect the informed consent format has on the preoperative anxiety of patients.

## Material and Methods

We have studied a total of 91 patients. A case is defined as follows: patient who will undergo inferior third molar surgery in the Service of Oral and Maxillofacial Surgery of the University Hospital Virgen del Rocío and who do not meet each and every one of the exclusion criteria of the study and who have voluntarily decided to take part in it.

The above mentioned exclusion criteria are: patients who have undergone a previous oral surgical procedure; patients with altered and/or impaired cognitive or communicative ability; patients with a previous history of anxiety episodes and anxiolytic treatment; patients whose questionnaires show some errors. Patients showing anxiety as a personality trait (this aspect will be assessed through the STAI inventory).

Once the patients are selected, they fill out the Spielberger State-Trait Anxiety Inventory – State (STAI-S). The STAI-S is a 20-item self-evaluation questionnaire, scored using a 4-level frequency scale ranging from 0 to 3 (maximum score: 60), that assesses transient emotional states or conditions as characterized by subjective feelings of tension and apprehension that can vary in time and intensity. We also collected information about the social profile of patients (occupation, age, sex and marital status). The distribution of patients into the different groups (written, oral or vide recording format) was completely at random following the order of intervention.

The sample under study was divided into three groups. The first group received oral information both about the procedure and the postoperative period and about the possible risks. The information provided was the same as that provided in the written document, which was given to the second group. All patients’ questions and queries were elicited by the physician.

The second group of patients received the same information as the first group but in written format. Also, all patients’ questions were answered.

The third group watched a video showing the surgical removal of an inferior third molar while an off voice explained it giving the same information that had been provided to the second group in written format. Once again, physicians answered patients’ questions.

Patients were then asked about their level of anxiety (0 calm, 1 somewhat anxious, 2 rather anxious, 3 very anxious), and whether the information provided had improved their level of anxiety or not and to what extent (from -3 much more anxious to +3 much more calm).

Data collected were entered into an MS-Excel data table (Microsoft Corp.- US) and exported to SPSS for Windows v.11 (SPSS Inc. – US). Normality of data was confirmed using the Kolmogorov-Smirnov test; the means of every group were compared using Student’s T test, ANOVA test and Bonferroni test. Qualitative variables were compared using the Chi-squared test. The study was approved by the Ethical Committee of the University of Seville and respected the Declaration of Helsinki.

## Results

A total of 134 patients were enrolled in the study, discarding 33 of them as they did not meet the inclusion criteria (anxiety was a personality trait in most of these patients). The final sample included 91 patients: 30 were assigned to the first group (oral informed consent), 31 to the second group (written informed consent) and another 30 to the third group (video recording informed consent).

Data regarding sex, age, marital status, occupation and anxiety level at onset of the study are shown in  Table 1. No statistically significant differences were observed between groups.

**Table 1:**
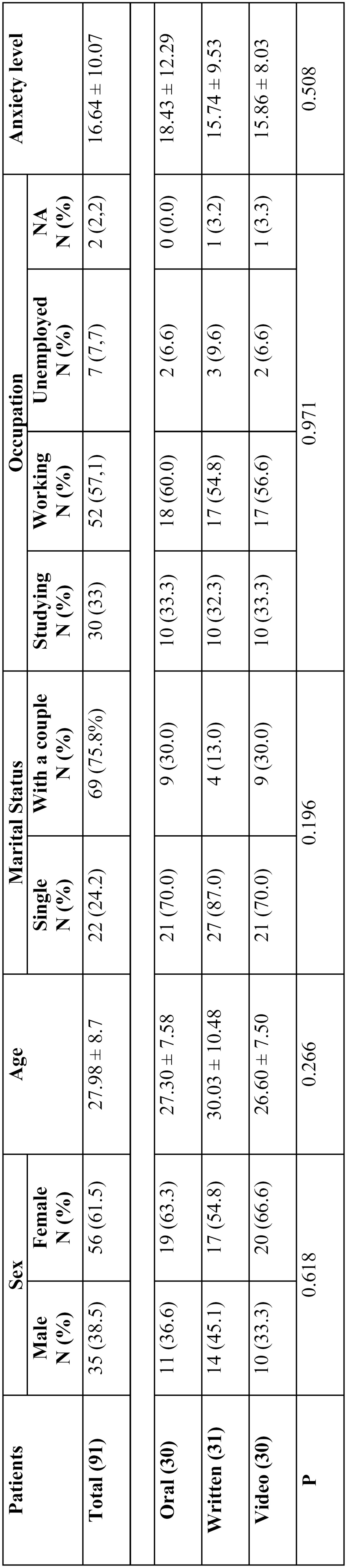
Characteristics of the sample before the informed consent.

Once the information had been provided to patients, the third group showed the highest anxiety level (video recording; 1.50±0.82) vs. group 1 (oral; 1.23±0.89) and group 2 (1.12±0.80). Nevertheless, differences were not statistically significant. ( Table 2).

**Table 2:**
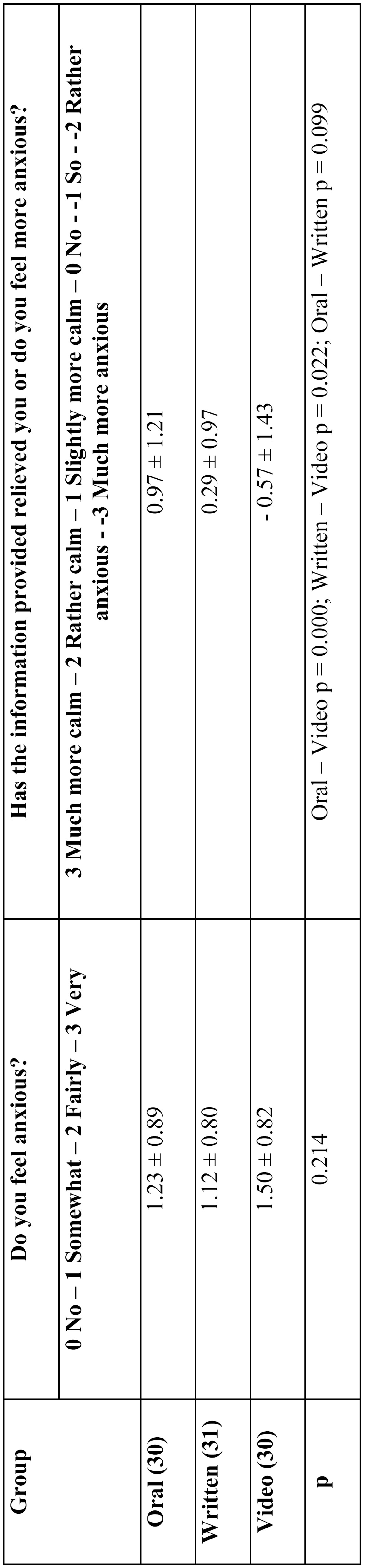
Characteristics of the sample after the informed consent.

But, whereas the oral and written information relieved patients to some extent (0.97±1.21 and 0.29±0.97, respectively) the video recording increased patients’ anxiety (0.57±1.43) in a statistically significant way. The difference between values corresponding to written and oral information was not statistically significant ( Table 2).

## Discussion

The aim of the informed consent cannot only be to comply with what is established by the law. It must be a process of communication between the oral surgeon and the patient which is the basis of a relationship based on trust. This will in turn improve the quality of the assistance the patient receives by diminishing his anxiety through the information provided (3).

This study is a step further in the attempt to improve medical attention by using an adequate informed consent format, analysing the effect of the different formats in which the information is provided on patients’ anxiety.

It has been previously established that the type of format is a key factor as regards patients’ satisfaction, comprehension, information recalling and decision to accept the treatment or not to do it (3).

Without underestimating the format itself, the main element to take into account in any informed consent is its content. By content we mean the content of the informed consent which must always convey two types of information. The first type refers to the treatment or procedure the patient will undergo. That is, the patient is informed about the different stages in the process: pre, intra and postoperative. The second type of information deals with the sensations the patient will probably experience: pain, somnolence, rigidity, etc.

It has been reported that the efficacy of informative techniques depends, to a great extent, on the attitude of patients (4,5). The information provided has been proved to have positive effects on those patients called “vigilant” (they try to overcome stressful situations by obtaining the maximum information possible about them), but may have negative effects on “avoidant” patients (they reject any sort of information trying thus to overcome anxiety by not thinking about the problem).

This is the reason why, by means of the present study, we have tried to homogenize in the best way possible the characteristics of the sample under study in relation with a secondary but relevant point of the informed consent: its format. Using the STAI inventory, whose reliability and validity is well-known and accepted, we have obtained the anxiety levels of our patients both as a personality trait (exclusion criterion) and as a particular state under a particular circumstance.

We agree with other researchers that the information provided to the patients as regards the intervention does not increase anxiety levels, as was previously thought (4). A study on 220 patients hospitalized before surgery shows that 82.3% of them did not feel more anxious after they had received the medical information (6). A recent study reveals that 57.7% of patients affirm that being informed about the intervention they were about to undergo did not increase their anxiety. In our study, patients were slightly anxious before the intervention, but the information provided had a relieving effect in two of the three groups.

Other studies show that the information has positive effects on “vigilant” patients (those who overcome stressful situations by obtaining as much information as possible about the origin of the stress), but not in the case of “avoidant” patients (those who reject information and try not to think about what is going to happen). It seems that the most appropriate is to provide patients with the information they require and in an adequate format (4,5).

In this sense, the most adequate format, according to our study, would be the oral interview between patient and physician, although we cannot state with statistical reliability that this format is better than the written format. Thus, it seems reasonable to suggest that the informed consent should be obtained through a mixed format (oral- written) which would also make it possible the contact between patient and physician.

The video recording format in the field of surgical procedures must be avoided as it increases patients’ anxiety and it does not show advantages for patients, which may exist in other fields of Dentistry as is the case of Cosmetic Dentistry.
